# Current obesity was associated with hypertension in children born very low birth weight

**DOI:** 10.1186/s13104-021-05611-x

**Published:** 2021-05-21

**Authors:** Chompoonut Limratchapong, Pracha Nuntnarumit, Wischuri Paksi, Kwanchai Pirojsakul

**Affiliations:** 1 Department of Pediatrics, Faculty of Medicine Ramathibodi Hospital, Mahidol University, Bangkok, Thailand; 2 Division of Nephrology, Department of Pediatrics, Ramathibodi Hospital, Mahidol University, Bangkok, Thailand

**Keywords:** Masked hypertension, Preterm, Very low birth weight, Ambulatory blood pressure monitoring, Obesity

## Abstract

**Objectives:**

Previous studies from the developed countries showed that children born very low birth weight have a higher risk of hypertension compared with that of the normal birth weight controls. However, studies regarding the prevalence of hypertension in such children from the developing countries are scarce. This study aimed to identify the perinatal and postnatal factors associated with hypertension in children born very low birth weight.

**Results:**

Forty-six children aged ≥ 6 years from the VLBW cohort of Ramathibodi Hospital, Bangkok, Thailand underwent the ambulatory blood pressure monitoring. The prevalence of hypertension was 15.2% (7/46). The hypertension group had a significant higher BMI z-score at 3 years of age (0.90 ± 1.44 vs − 0.45 ± 1.47, *p* = 0.045) and a greater proportion of current obesity (42% vs 2.5%, *p* < 0.01) compared to those in the normotensive group. Multivariate analysis revealed that current obesity was associated with hypertension (OR 34.77, 95%CI 1.814–666.5). Among 36 children with normal office blood pressure, four children (11.1%) had high blood pressure uncovered by ABPM, called “masked hypertension”. Office systolic blood pressure at the 85th percentile was the greatest predictor for masked hypertension with a sensitivity of 75% and a specificity of 81.2%.

## Introduction

The World Health Organization estimated the prevalence of preterm birth to be 5–18% across 184 countries and the preterm birth rates have been increasing in almost all countries [1]. Advances in perinatal medicine have led to a continuous decrease in the mortality of these children [2]. In preterm survivors, there is a high rate of long-term complications such as recurrent hospitalizations, long-term neurodevelopmental impairment, and chronic health problems, including hypertension and vascular change. Previous studies have shown that preterm with low birth weight is a risk factor for higher office blood pressure compared with that of the term and normal birth weight controls [3–6].

Patients who have normal office blood pressure but elevated out-of-office blood pressure are called having “masked hypertension” [7]. Compared to normotensive children, children with masked hypertension have a significant risk for end-organ damage [8–10]. Ambulatory blood pressure monitoring (ABPM) can uncover masked hypertension in children with normal office blood pressure, detect white coat hypertension and confirm hypertension in children with elevated office blood pressure. Among children and adolescents, the estimated prevalence of masked hypertension ranged from 7.4 to 11.0% [11, 12]. Previous studies have shown that children born very low birth weight (VLBW) had higher both awake and sleep BP with reduced nocturnal dipping compared with those of the normal birthweight individuals [4, 13–15]. This study was aimed to assess office and ambulatory blood pressure in children born VLBW and to identify perinatal, postnatal and present clinical parameters associated with hypertension. In addition, we aimed to identify the parameter for the prediction of masked hypertension in children who had normal office blood pressure.

## Main text

### Methods

Children aged ≥ 6 years from the cohort of children born VLBW (< 1500 g) at Ramathibodi Hospital, Bangkok, Thailand were enrolled. Children who had chronic diseases that may contribute to the development of hypertension e.g. known kidney diseases, etc., or who were not contactable at the time of recruitment were excluded. Detailed perinatal data were collected including gestational age, birth weight, maternal hypertension during pregnancy, antenatal steroid therapy, umbilical catheter insertion, and other perinatal problems such as respiratory distress syndrome, bronchopulmonary dysplasia, intraventricular hemorrhage, etc. The birth weight z-score was calculated according to the Fenton growth for preterm infants [16, 17]. Being small for gestational age was defined as a z-score less than − 2 for sex and gestational age. Patients from the VLBW cohort had been assessed postnatal weight and height since birth to at least 3 years of age, their ages were analyzed with correction for prematurity. The data at time of enrollment were collected including demographic data (sex, age); anthropometric data (weight, height, body mass index (BMI), waist circumference); laboratory values (serum creatinine, estimated glomerular filtration rate (eGFR) estimated by the bedside Schwartz formula [18] and urinalysis). Weight, height, and BMI were normalized to z-score using age- and sex-specific formulas provided by the Centers of Disease Control and Prevention [19]. Obesity is defined as a BMI z-score ≥ 2. Waist circumference-height ratio was calculated using waist circumference (cm)/height (cm).

Measurements of office blood pressure were performed by oscillometric method using Dinamap™ V100 (GE Healthcare, Chicago, Illinois, USA). The device was applied in the right arm with a corrected cuff size after being rest for 5 min in the sitting position with feet on the floor and arm and back supported. Readings were taken three times after resting for five minutes between measurements and the average of the three measurements was used for analysis. Z-scores of office blood pressure were calculated based on the normative pediatric BP tables of normal-weight children by the American Academy of Pediatrics [7]. Office hypertension was defined as systolic blood pressure (SBP) and/or diastolic blood pressure (DBP) z-score ≥ 95th percentile adjusted for sex, age, and height.

ABPM was performed with the A&D TM-2430 device (A&D, Tokyo, Japan), which has been validated for use in children and adolescents [20]. Measurements were performed every 20 min during awake and every 30 min during sleep. Patients were instructed to perform their routine activities, avoid vigorous activities and to record their daily activities and time they woke up and went to sleep. ABPM was considered adequate if there were ≥ 40 valid BP readings for the entire 24-h period. Sustained hypertension is defined as having a high office blood pressure and mean ambulatory SBP or DBP ≥ the 95th percentile for gender and height and blood pressure load ≥ 25% either awake, sleep or both periods according to the current guidelines [21]. Masked hypertension is defined as having a normal office BP but high ambulatory blood pressure as defined previously. Whitecoat hypertension is defined as having a high office blood pressure but normal ambulatory blood pressure.

The IBM SPSS^®^ software version 22 was used for analysis. Data were presented as percentages, mean and standard deviation or median and interquartile range (IQR), as appropriate. Categorical data were compared using the chi-square test or Fisher’s exact test, as appropriate. Student’s t-test or Mann–Whitney U test was used for comparison of continuous data between the two groups, as appropriate. Factors associated with hypertension were analyzed by univariate logistic regression and the potential significant parameters (*p* ≤ 0.2) were selected to be analyzed with multivariate analysis. The receiver operating characteristic (ROC) curve was used to analyze the performance of the parameters for detecting masked hypertension. A *p* ≤ 0.05 was considered statistically significant.

### Results

The data of all patients born VLBW in the pediatric department during the years 2004 and 2012 were reviewed. From this population (402 patients), only 110 patients were contactable at the time of recruitment due to changes in the address and telephone numbers. Of these, 37 patients were living in the other regions and 10 patients refused to participate in this study. Seventeen patients were excluded from the study due to severe BPD (1), obstructive sleep apnea (4), known chronic kidney diseases (3), central nervous system anomalies (2), and attention deficit hyperactivity disorder who received psychostimulants (7). Therefore, 46 patients (19 males) participated in this study. Baseline characteristics of all participants were summarized in Table 1.

**Table 1:** Perinatal conditions and postnatal growth between patients with and without hypertension

Parameters	All patients (N = 46)	Hypertension (N = 7)	No hypertension (N = 39)	*P*-value
Perinatal conditions				
SGA, n (%)	10 (21)	1 (14)	9 (23)	0.604
Mode of delivery, C/S, n (%)	31 (67)	3 (42)	28 (71)	0.193
GA (weeks), mean ± SD	29.70 ± 2.88	30.14 ± 2.41	29.62 ± 2.97	0.661
Birth weight (g), mean ± SD	1143 ± 278	1237 ± 227	1127 ± 286	0.341
Maternal preeclampsia, n (%)	15 (33)	2 (28)	13 (33)	0.807
Antenatal steroid used, n (%)	36 (78)	4 (57)	32 (82)	0.146
RDS, n (%)	26 (56)	4 (57)	22 (56)	0.971
TTNB, n (%)	7 (15)	1 (14)	6 (15)	0.941
BPD, n (%)	20 (43)	3 (42)	17 (43)	0.971
PDA, n (%)	18 (39)	2 (28)	16 (41)	0.534
IVH, n (%)	10 (22)	2 (28)	8 (20)	0.634
NEC, n (%)	5 (11)	0 (0)	5 (12)	0.316
Sepsis, n (%)	11 (24)	1 (14)	10 (25)	0.517
Aminoglycoside used, n (%)	34 (74)	6 (85)	28 (71)	0.589
TPN transfusion, n (%)	36 (78)	5 (71)	31 (79)	0.752
Invasive ventilator used, n (%)	17 (37)	1 (14)	16 (41)	0.177
UAC insertion, n (%)	28 (61)	4 (57)	24 (61)	0.809
UVC insertion, n (%)	32 (70)	4 (57)	28 (71)	0.796
Length of stay (days), mean ± SD	59 ± 29	54 ± 28	60 ± 29	0.600
Discharge weight (g), mean ± SD	2619 ± 634	2662 ± 830	2612 ± 606	0.851
Postnatal growth				
BMI z-score at 2 years old, mean ± SD	− 0.37 ± 1.20	0.38 ± 0.97	− 0.52 ± 1.19	0.090
BMI z-score at 2.5 years old, mean ± SD	− 0.45 ± 1.27	0.36 ± 1.07	− 0.58 ± 1.26	0.126
BMI z-score at 3 years old, mean ± SD	− 0.24 ± 1.54	0.90 ± 1.44	− 0.45 ± 1.47	**0.045**

Bold font indicates statistical significance

*BPD* bronchopulmonary dysplasia, *C/S* cesarean section, *GA* gestational age, *IVH* intraventricular hemorrhage, *NEC* necrotizing enterocolitis, *PDA* patent ductus arteriosus, *RDS* respiratory distress syndrome, *SD* standard deviation, *SGA* small for gestational age, *TPN* total parenteral nutrition, *TTNB* transient tachypnea of the newborn, *UAC* umbilical artery catheter, *UVC* umbilical venous catheter

Office blood pressure and ABPM were performed in all participants. Regarding office blood pressure, 10 patients of 46 participants (21.7%) had office hypertension. Among patients with office hypertension, 3 patients had hypertension confirmed by ABPM so-called “sustained hypertension”, while another 7 patients had normal ambulatory blood pressure so-called “white-coat hypertension”. Regarding 36 patients with normal office blood pressure, 4 children (11.1%) had masked hypertension uncovered by ABPM. Therefore, the overall prevalence of hypertension was 15.2% (7/46).

Comparisons of perinatal parameters and postnatal growth between patients with and without hypertension were presented in Table 1. The postnatal growth data showed that the group of patients with hypertension had a significantly higher mean BMI z-score at 3 years old compared to that of the normotensive group (0.90 ± 1.44 vs − 0.45 ± 1.47, *p* = 0.045). Comparisons of the current anthropometric parameters and laboratory investigation between the groups with and without hypertension were presented in Table 2. The hypertensive group had a significant greater proportion of obesity (42% vs 2.5%, *p* < 0.01) and higher WHR (0.51 ± 0.06 vs 0.46 ± 0.05, *p* = 0.023) compared to those in the normotensive group. Multivariate analysis adjusted for birth weight and antenatal steroid exposure and birth weight showed that current obesity was a significant factor associated with hypertension (OR 34.77, *p* = 0.019 95%CI 1.814–666.5) as shown in Table 3.

**Table 2:** Current demographic data, anthropometric data and laboratory investigation between hypertensive and normal groups

Parameters	Hypertension (N = 7)	No hypertension (N = 39)	*P*-value
Current demographic data			
Gender, male, n (%)	3 (42)	16 (41)	0.611
Age (years) mean ± SD	8.57 ± 2.14	9.10 ± 2.58	0.928
Current anthropometric data			
Body weight (kg), mean ± SD	34.55 ± 9.14	32.39 ± 13.14	0.680
Body weight z-score, mean ± SD	0.79 ± 1.84	0.03 ± 1.55	0.256
Height (cm), mean ± SD	133.24 ± 7.14	134.38 ± 16.65	0.860
Height z-score, mean ± SD	0.47 ± 1.31	0.09 ± 1.25	0.469
BMI z-score, mean ± SD	0.70 ± 1.75	− 0.24 ± 1.77	0.200
Obesity, n (%)	3 (42)	1 (2.5)	** < 0.01**
Waist-height ratio (cm/cm), mean ± SD	0.51 ± 0.06	0.46 ± 0.05	**0.023**
Laboratory investigation			
Serum creatinine (mg/dL), mean ± SD	0.47 ± 0.07	0.53 ± 0.11	0.211
eGFR (mL/min/1.73 m^2^), mean ± SD	117 ± 16.42	107 ± 22.84	0.316
Urinary protein positivity, n (%)	0 (0)	1 (2.56)	0.692

Bold font indicates statistical significance

*BMI* body mass index, *eGFR* estimated glomerular filtration rate, *SD* standard deviation

**Table 3:** Association of perinatal and postnatal conditions and childhood hypertension

Parameters	Univariate analysis			Multivariate analysis		
	Exp (β)	*P*-value	95%CI	Exp (β)	*P*-value	95%CI
Current obesity	**28.5**	**0.008**	**2.37–342.59**	**34.77**	**0.019**	**1.814–666.5**
Mode of delivery, C/S	0.295	0.147	0.057–1.536	3.702	0.203	0.493–28.822
Antenatal steroid used	0.292	0.157	0.053–1.606	1.454	0.773	0.115–18.374
SGA	0.556	0.608	0.059–5.241			
GA	1.066	0.563	0.807–1.408			
BW	1.002	0.339	0.998–1.005			
Maternal preeclampsia	0.8	0.805	0.136–4.696			
RDS	1.03	0.971	0.203–5.234			
TTNB	0.917	0.941	0.093–9.041			
BPD	0.971	0.971	0.191–4.93			
PDA	0.575	0.538	0.099–3.341			
IVH	1.55	0.636	0.252–9.516			
Sepsis	0.483	0.524	0.052–4.521			
Aminoglycoside used	1.286	0.83	0.13–12.738			
TPN transfusion	0.565	0.541	0.09–3.537			
Invasive ventilator used	0.24	0.205	0.026–2.186			
UAC insertion	0.8	0.81	0.13–4.917			
UVC insertion	0.786	0.797	0.125–4.923			
Length of stay	0.992	0.593	0.963–1.022			
Discharge weight	1	0.847	0.999–1.001			
Gender, male	1.078	0.928	0.212–5.488			
Age	0.915	0.604	0.654–1.28			
Body weight z-score	1.387	0.253	0.791–2.431			
Height z-score	0.995	0.856	0.944–1.049			
BMI z-score	1.54	0.201	0.795–2.983			
Serum creatinine	0.001	0.205	0.0–46.0			
eGFR	1.019	0.314	0.982–1.057			

Bold font indicates statistical significance

Regarding 36 children with normal office blood pressure, 4 patients with masked hypertension had a significantly higher mean office SBP z-score than that of the group without masked hypertension (1.10 ± 0.35 vs 0.32 ± 0.73, *p* = 0.01). The ROC analysis showed that SBP z-score was the best predictor of masked hypertension with the area under the curve of 0.85. The office SBP cutoff level at the 85th percentile had a sensitivity of 75% and a specificity of 81.2%.

### Discussion

The present study revealed that the overall prevalence of hypertension was 15.2% and the current obesity was the factor associated with hypertension. Moreover, office SBP above the 85th percentile was a good office BP parameter for the prediction of masked hypertension in children born VLBW who had normal office blood pressure.

The overall prevalence of hypertension in this cohort was higher than that of the previous reports of hypertension in children born preterm and VLBW, ranging from 6 to 13% [5, 6, 22]. This discordant finding could be explained by the fact that the present study used ABPM that can detect masked hypertension, while the previous studies used only the office blood pressure measurements. On the other hand, the prevalence of masked hypertension in this study was 8.6%, similar to that reported globally, which estimated as 9–16% among the pediatric population [11, 12, 23–25]. Not only sustained hypertension but also masked hypertension is associated with the increase in carotid intimal media thickness and adverse cardiovascular structure in children and adolescents [8, 10, 26]. This finding supports the current clinical practice guidelines that suggest using ABPM for hypertension screening in patients born prematurity and low birth weight [7].

Our study demonstrated an association between current obesity and hypertension in children born VLBW which was consistent with the previous studies. The study from the USA found that obesity was significantly associated with hypertension and the rate of weight gain since birth among extremely LBW infants were associated with an increase in SBP [22]. Lurbe et al. reported that all of the office, 24-h, daytime and nighttime SBP were significantly higher in LBW Spanish children who became later obese [27]. In addition, the present study found that the BMI z-score at 3 years of age was significantly higher in the group with hypertension than that of the group without hypertension. Lule et al. reported that the accelerated weight gain from birth to 6 months of age was associated with hypertension in the 2nd decade of life in LBW patients [28]. These findings support that accelerated postnatal growth is associated with childhood hypertension. The other studies also reported maternal preeclampsia and antenatal steroid as the additional risk factors for hypertension [5, 6]. However, this study showed no association between these factors and hypertension. This might be the result of a small number of participants in the present study.

The present study identified that the cutoff level of office SBP at the 85th percentile had the highest sensitivity (75%) and specificity (81.2%) to predict masked hypertension. Hamdani G et al. reported that the 85th percentile of SBP had a sensitivity of 86.8% and a specificity of 57.4% to diagnose ambulatory hypertension in healthy adolescents [29]. As same as the study of Centra et al., office SBP measurement of ≥ 122.5 mmHg that was equal to the 85th percentile of SBP predicted masked hypertension in adults born extreme preterm and extremely LBW with a sensitivity and a specificity of 79% and 74%, respectively [3]. Interestingly, our study found that the prevalence of white-coat hypertension was relatively high at 70% (7/10) and it was higher than previous reports in previously healthy children and adolescents, which estimated 34–58% [10, 11, 30, 31]. Therefore, using ABPM to exclude white-coat hypertension also might be useful in children born VLBW, which could prevent unnecessary investigation and use of anti-hypertensive medication.

## Conclusion

The prevalence of hypertension was 15.2% in this cohort of children born VLBW. The current obesity was an independent factor associated with hypertension during the childhood period.

## Limitations

The present study had some limitations. The sample size was small, especially the proportion of children with masked hypertension. This might not have enough power to detect some differences of the parameters between the groups with and without hypertension. Due to a retrospective design with a cross-sectional survey, incomplete data existed. Office BP was measured on only one visit, therefore it might not correctly define office hypertension. Lastly, there was no available standard ambulatory blood pressure tables for Thai children, so the authors used a normogram reported by the European investigators [32].

## Data Availability

Data will be obtained up on a reasonable request by emailing to the corresponding author using “kwanchai.pio@mahidol.ac.th”.

## Abbreviations

ABPM: Ambulatory blood pressure monitoring
VLBW: Very low birth weight
BPD: Bronchopulmonary dysplasia
C/S: Cesarean section
GA: Gestational age
IVH: Intraventricular hemorrhage
NEC: Necrotizing enterocolitis
PDA: Patent ductus arteriosus
RDS: Respiratory distress syndrome
SD: Standard deviation
SGA: Small for gestational age
TPN: Total parenteral nutrition
TTNB: Transient tachypnea of the newborn
UAC: Umbilical artery catheter
UVC: Umbilical venous catheter
BMI: Body mass index
eGFR: Estimate glomerular filtration rate
SD: Standard deviation
